# Reopenable clip-over-the-line method with endoloop anchoring for gastric defect closure after endoscopic submucosal dissection

**DOI:** 10.1055/a-2884-9287

**Published:** 2026-06-12

**Authors:** Tomohiko Mannami, Tsuyoshi Umekawa, Taichi Kase, Yasushi Fukumoto, Hanako Nagahara, Tsukasa Sakaki, Shin'ichi Shimizu

**Affiliations:** 1 Department of Gastroenterology 73745 NHO Okayama Medical CenterOkayamaOkayama PrefectureJapan

Large mucosal defects after endoscopic submucosal dissection (ESD) increase the risk of delayed adverse events. 1​ A reopenable clip-over-the-line method (ROLM) is an effective technique that enables secure closure of such defects. 2​ ​3​ Stability of the closure is maintained by the frictional force between the line, the clip, and the surrounding tissue. 4​ This led us to consider the use of anchoring at the end of the ROLM line to enhance the stability further. Thus, we present a modified method—ROLM with endoloop anchoring (ROLM-e)—incorporating an additional fixation step in the standard ROLM.

**Fig. 1:**
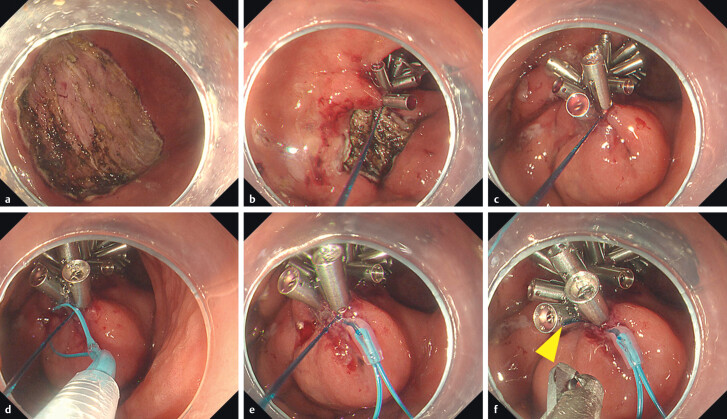
Endoscopic images demonstrating the reopenable clip-over-the-line method with endoloop anchoring (ROLM-e) in a representative case. ( **a** ) A 35 × 30-mm mucosal defect at the greater curvature of the gastric body after endoscopic submucosal dissection. ( **b** and **c** ) The defect is closed using the reopenable clip-over-the-line method (ROLM) with reopenable clips (SureClip; 16 mm; MC Medical, Tokyo, Japan) and a 0.23-mm nylon line. ( **d** ) After completing the ROLM, a detachable snare (HX-400U-30; Olympus Medical Systems, Tokyo, Japan) is advanced after passing the ROLM line through the endoloop outside the endoscope. ( **e** ) The endoloop is tightened to secure the line adjacent to the final clip, establishing endoloop anchoring. ( **f** ) The final appearance after cutting the externalized line, leaving approximately 1 cm of the line. The arrowhead indicates the cut end of the line.

**Fig. 2:**
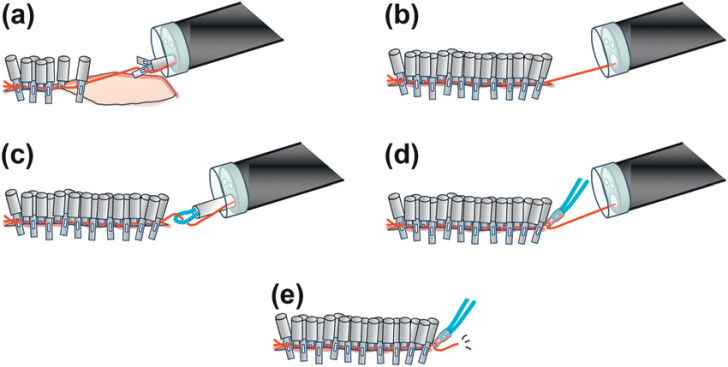
Schematic illustration of the reopenable clip-over-the-line method with endoloop anchoring (ROLM-e). ( **a–b** ) Closure using the standard reopenable clip-over-the-line method (ROLM) is performed by sequentially placing the reopenable clips along a traction line to approximate the mucosal defect. ( **c** ) After completing the ROLM, a detachable snare is advanced through the accessory channel and positioned at the base of the line emerging from the final clip. ( **d** and **e** ) The snare is tightened to achieve endoloop anchoring, providing additional fixation before cutting the externalized line.

An 85-year-old woman underwent gastric ESD for adenocarcinoma, resulting in a 35×30-mm mucosal defect. While performing ROLM, a reopenable clip (SureClip, 16 mm; MC Medical, Tokyo, Japan) with an attached 0.23-mm nylon line was placed at one edge of the defect, and the line was sequentially passed through additional clips positioned along the defect’s opposite side. Repeating this process (23 clips in total) gradually approximated the defect from the anal to the oral side ( Fig. 1 ). After completing ROLM, endoloop anchoring was performed using a detachable snare (HX-400U-30; Olympus Medical Systems, Tokyo, Japan). Before insertion, the loop was pre-tightened to a small diameter and introduced through the accessory channel ( Fig. 1 ). The loop was subsequently positioned at the base of the line emerging from the final ROLM clip and tightened to achieve endoloop anchoring. Finally, the line’s external end was cut to complete the procedure ( Fig. 2 ). Endoscopic follow-up performed on postoperative days 1 and 7 confirmed that the endoloop anchoring remained intact in this case and in two additional consecutive cases ( Video 1 ). No intra- or post-procedural adverse effect was observed in all three cases.

**Video 1** The reopenable clip-over-the-line method with endoloop anchoring (ROLM-e) for gastric defect closure after endoscopic submucosal dissection. After completing the standard ROLM, the endoloop is tightened to secure the line adjacent to the final clip.


Secure closure of mucosal defects is a persistent challenge, and in suturing procedures other than ROLM, anchoring techniques such as the usage of felt pledget pieces have been proposed. 5​ To the best of our knowledge, this is the first report describing the addition of anchoring to ROLM using a commercially available endoloop that offers a straightforward and cost-effective option. ROLM-e is a simple modification that can enhance closure stability after ESD. This approach could be especially useful in situations with potentially reduced frictional forces, such as in smaller defects requiring fewer clips, when fewer clips are intentionally used to simplify or expedite the procedure, or during the early learning phase, when closure durability may be more susceptible to technical factors.

Endoscopy_UCTN_Code_TTT_1AO_2AO
